# Nanoengineered Self-Assembling Peptides with Increased Proteolytic Stability Promote Wound Healing

**DOI:** 10.1021/acsami.4c18221

**Published:** 2025-02-12

**Authors:** Vânia I. B. Castro, Ana Rita Araújo, Rui L. Reis, Iva Pashkuleva, Ricardo A. Pires

**Affiliations:** † 3B’s Research Group, I3Bs − Research Institute on Biomaterials, Biodegradables and Biomimetics, University of Minho, Headquarters of the European Institute of Excellence on Tissue Engineering and Regenerative Medicine, 4805-017 Barco, Portugal; ‡ ICVS/3B’s−PT Government Associate Laboratory, 4805-017 Braga/Guimarães, Portugal

**Keywords:** self-assembly, bioactive peptides, GHK, wound healing, enzymatic degradation

## Abstract

The copper complex of the tripeptide glycine–histidine–lysine (GHK) has proven benefits in wound healing and tissue remodeling by promoting blood vessel growth and increasing skin oxygen levels, but its activity is reduced in body fluids due to fast proteolytic cleavage. Herein, we designed several peptides that bear the GHK sequence and can self-assemble into supramolecular nanostructures aiming for enhanced bioactivity. The design involves a phenylalanine (F) backbone known for its ability to form supramolecular assemblies. We tested either coassembly between the structural peptide F_4_D and the functional sequence GHK or assembly of covalently bound peptides, in which the GHK is bound via the glycine (F_4_D-GHK) or lysine (F_4_D-KHG, i.e., inverted GHK sequence). All tested peptides assembled into nanotapes, but their resistance to proteolytic degradation was different: covalently bound peptides generated more stable assemblies. Wound healing assays demonstrated that the supramolecular structures have enhanced bioactivity when compared to GHK alone. Multiplex immunoassay analyses demonstrated the secretion of key regulators of the healing process, such as cytokines, matrix metalloproteinases, and growth factors. Altogether our data show that incorporation of GHK/KHG into supramolecular structures improves its stability, bioactivity, and efficacy in promoting wound healing.

## Graphical abstract

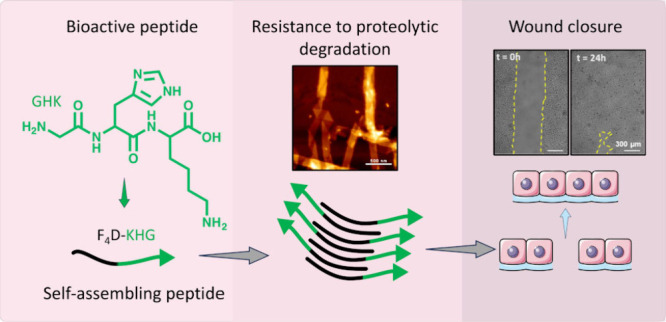

## 1 Introduction

Wound healing is a complex process of coordinated cellular and biochemical events triggered by tissue injury. This process typically involves three distinct but overlapping phases: (1) hemostasis/inflammation, (2) proliferation and migration, and (3) maturation/remodeling.^1,2^ During the first phase, the bleeding is ceased, and the inflammatory response is activated. It is characterized by increased vascular permeability, release of cytokines and growth factors, and activation of various cell types.^1,3^ In the second phase, fibroblasts migrate into the wound site, where they proliferate and secrete an extracellular matrix. In this phase, keratinocytes also migrate from the wound edges to cover the injured area and proliferate until the wound is fully covered. Angiogenesis, a process involving the migration, proliferation, and organization of vascular endothelial cells, occurs as well and leads to matrix formation and epithelialization. Finally, in the maturation and remodeling phases, the wound undergoes structural reorganization and strengthening. A failure or delay in any of these phases leads to prolongation and/or failure of the wound healing and thus presents a clinical challenge associated with increased healthcare costs and reduced patient quality of life. Because skin’s ability to heal declines with age, the impact of poor wound healing is even higher for the elderly population and prompts the need for effective solutions.

GHK (glycine–histidine–lysine) is a natural tripeptide found in human saliva, plasma, and urine, known for its benefits on wound healing.^4–7^ It is a product of the proteolytic degradation of various extracellular matrix proteins such as collagen. The released GHK complexes with Cu^2+^ and the formed GHK-Cu complex has several key functions in wound healing and skin regeneration by stimulating the synthesis of collagen and by modulating the activity of key metalloproteinases.^8–12^ It has also anti-inflammatory properties, reduces the oxidative stress by neutralizing free radicals, and influences the expression of genes involved in tissue repair.^7,13–15^ Moreover, GHK-Cu promotes angiogenesis: Cu^2+^ ions are crucial angiogenic factors, stimulating the migration and tube formation by human endothelial cells.^16,17^ Due to its properties, GHK-Cu is used as an additive to cosmetic products to increase the production of skin collagen, reduce skin irritation and redness, and mitigate age spots, photodamage, and hyperpigmentation.^4^

At physiological conditions, its activity is short due to rapid proteolytic degradation, and this effect is even more pronounced at some pathological conditions such as diabetic and venous stasis skin ulcers.^19^ Thus, different strategies have been used to protect GHK usually by its encapsulation into hydrogels^20^ and liposomes.^21^ Nanoprotein engineering is another approach to increase the stability and enhance the protein’s bioactivity. It merges the principles of nanotechnology with protein engineering to develop bioactive molecules with enhanced or novel properties for various applications in medicine, biotechnology, and materials science. In this work, we applied these principles to GHK by appending the GHK/KHG sequence onto F_4_D to allow nanostructure formation by self-assembly. We studied different peptide designs/assembly conditions: GHK was either unmodified and coassembled with F_4_D or covalently bound to F_4_D at the G (F_4_D-GHK) or at K (F_4_D-KHG) position. The copper-binding ability, stability, and bioactivity of the generated nanoassemblies were assessed and showed enhanced capacity to promote wound healing when compared to GHK-Cu in solution.

## 2 Results and Discussion

### 2.1 Peptide Design, Synthesis, and Self-Assembly

Our molecular design builds on previous data showing that GHK alone forms random aggregates but when coassembled with the tripeptide FFD generates nanotapes.^22,23^ The coassembly is driven by electrostatic interactions between K and D.^22^ However, our initial tests demonstrated that the stability of this system under physiological conditions is limited and compromises the generated nanoassemblies. Thus, we increased the number of the F residues in the structural peptide to 4 (i.e., F_4_D), aiming at higher peptide hydrophobicity and enhanced π-interactions between the phenyl groups that stabilize the nanostructure. The coassembly of F_4_D with GHK (Pep1, Figure 1A) was tested in the absence and in the presence of Cu^2+^.

**Figure 1:**
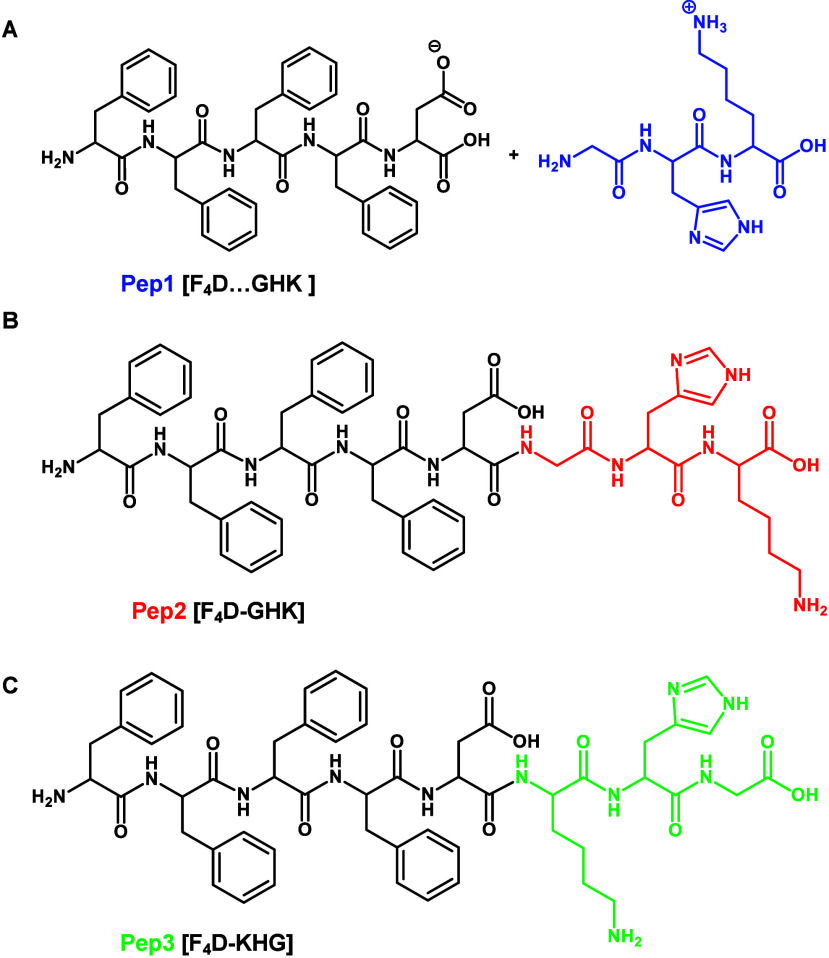
Chemical structures of the synthesized peptides: (A) Pep1 (coassembly between F_4_D and GHK); (B) Pep2 (F_4_D-GHK); and (C) Pep3 (F_4_D-KHG).

A synthetic analogue, in which F_4_D and GHK are covalently bonded (Pep2, Figure 1B) was also obtained and assessed. Because previous data has shown that Cu^2+^ binds at the glycine (G) and histidine sites (H) of GHK,^11,19^ we also designed and studied Pep3 (Figure 1C) in which the GHK sequence is inverted and the conjugation to the F_4_D is promoted at the lysine (K) site and not at the glycine (G) one. We hypothesize that the different spatial exposure G and H in this heptapeptide can enhance the complexation with Cu^2+^ and thus increase the bioactivity of Pep3 in comparison to Pep2.

All peptides were synthesized via automated solid-phase peptide synthesis. Their purity and structure were confirmed by high-performance liquid chromatography, mass spectroscopy, and nuclear magnetic resonance (Figures S1–S9). We used the pH-switch method to induce self-assembly, i.e., the peptides were dissolved at basic conditions (pH ≈ 10) and the assembly was triggered by a decrease of the pH to the physiological one. At these conditions, we observed the formation of nanotapes/nanofibers with different widths/diameters for Pep1 (∼220 nm), Pep2 (∼235 nm), and Pep3 (∼60 nm) (Figure 2A). The addition of Cu^2+^ induced significant changes in the morphology and width/diameter of the nanotapes/nanofibers, i.e., Pep1-Cu (∼80 nm), Pep2-Cu (150 nm), and Pep3-Cu (∼145 nm), indicating an ongoing complexation process (Figure 2B). Of note, we did not observe any nanostructures for GHK alone or in the presence of Cu^2+^ ions (Figure S10) at the studied conditions. On the other hand, the neutralization of an alkaline solution of F_4_D resulted in the formation of well-defined and unidirectional structures in the form of nanotapes/nanofibers as expected (Figure S11).

**Figure 2:**
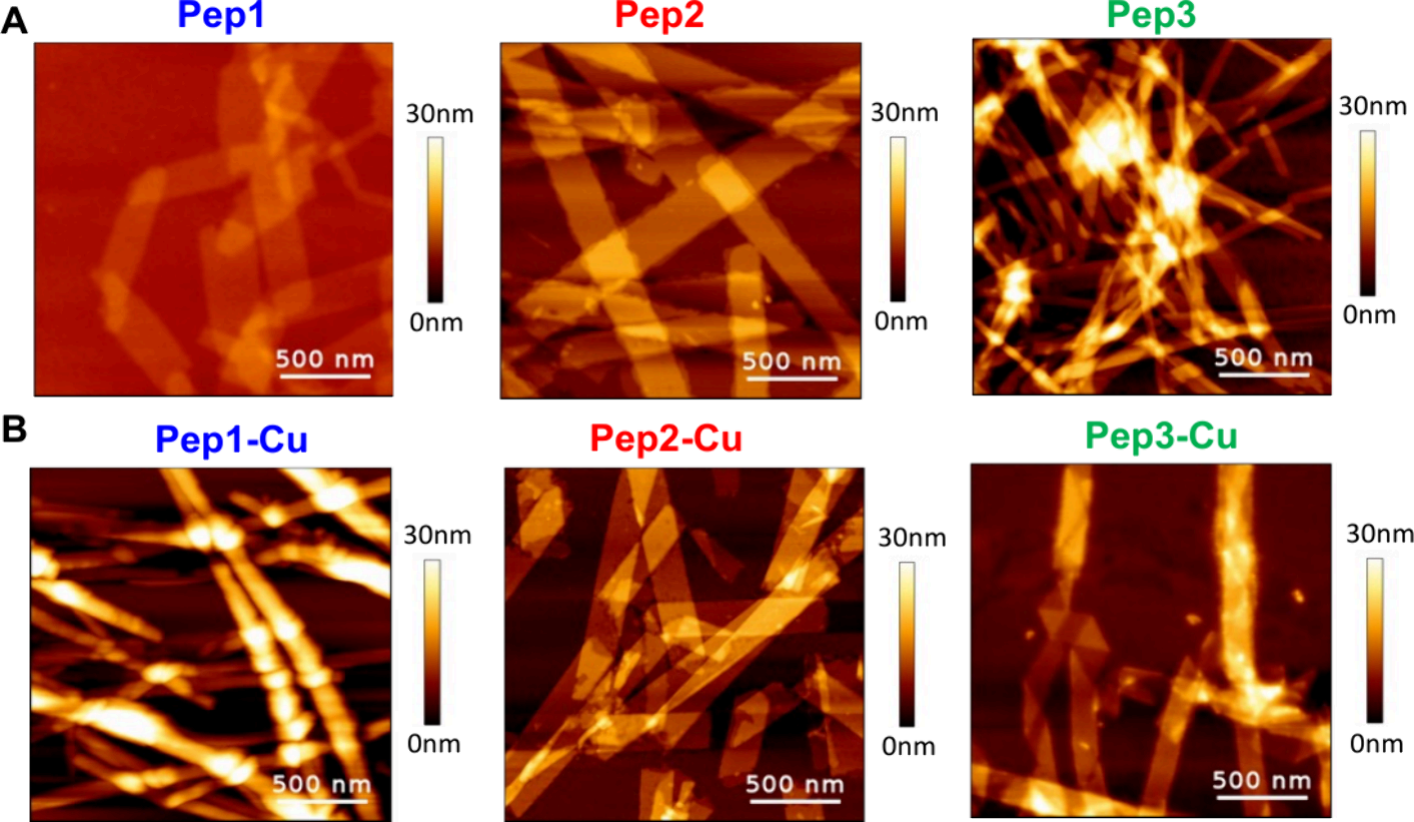
AFM images of the nanostructures assembled from the studied peptides by the pH switch method: (A) nanoassemblies formed in the absence of Cu^2+^ and (B) nanoassemblies formed in the presence of Cu^2+^. Scale bar = 500 nm. Control experiments with GHK and F_4_D alone are shown in Figures S10 and S11, respectively.

The circular dichroism (CD) spectra of the assemblies showed a β-sheet-like conformation for all peptides, as demonstrated by a positive peak around 205 nm and a negative one at 230 nm.^24^ While it is difficult to establish a direct connection between the secondary structure of the peptides and nanofiber/nanotape morphology, our CD and AFM results showed that the addition of Cu^2+^ induced changes in the supramolecular organization of the peptides (Figure 3A) and in their morphology, which is consistent with the formation of peptide–Cu^2+^ complexes.^13,25^

**Figure 3:**
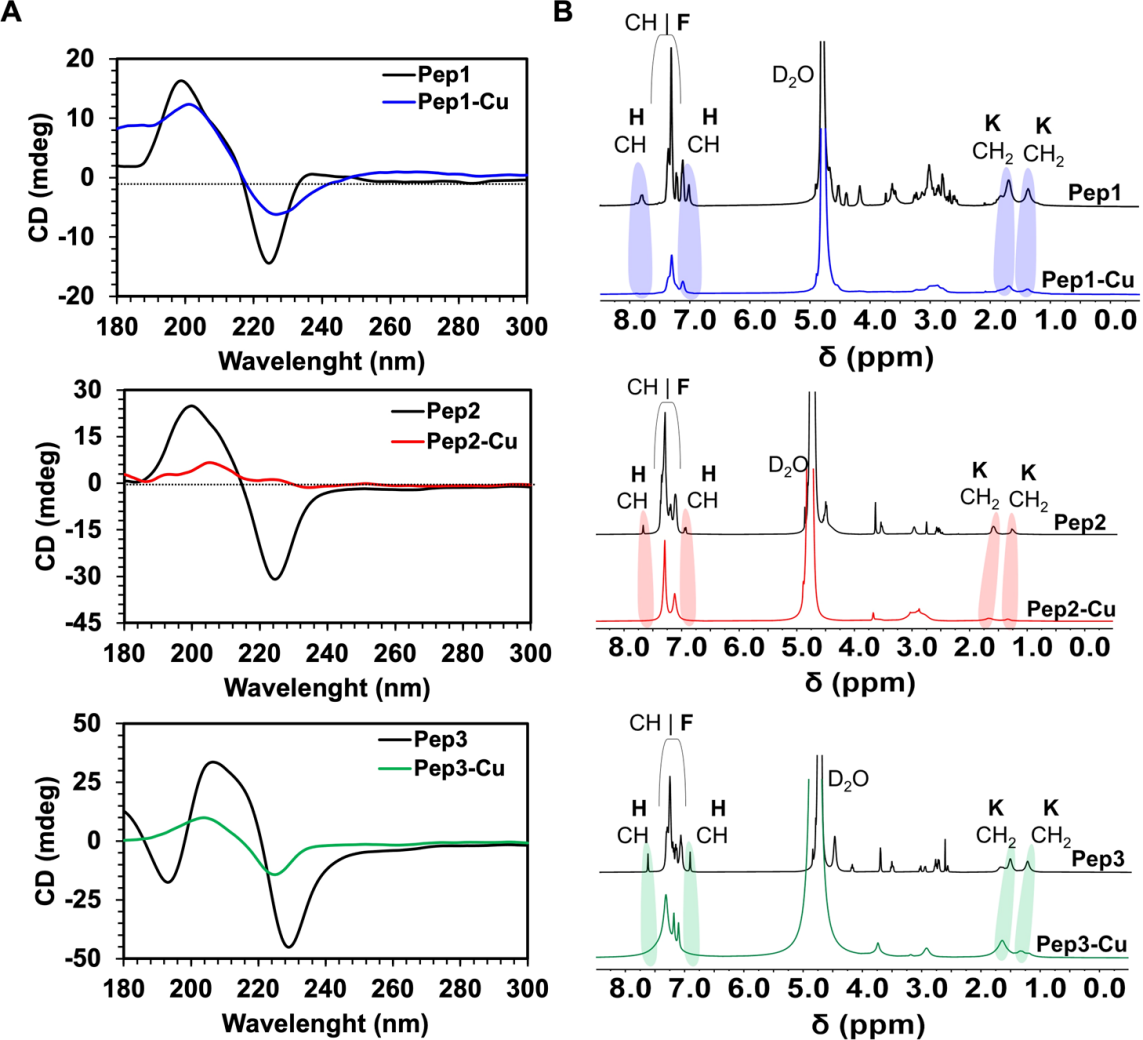
Spectroscopic characterization of the supramolecular structures assembled from the studied peptides in the presence (black line) and absence (colored line) of Cu^2+^: (A) CD spectra and (B) ^1^H NMR spectra of the assemblies generated by Pep1–Pep3 showing the impact of the Cu^2+^ on the molecular structure of the generated assemblies.

Nuclear magnetic resonance (NMR) spectra complemented these data. Liquid state ^1^H NMR is sensitive to molecular mobility, and a restriction in movement often leads to a broadening (or disappearance) of the NMR signals. The signals for histidine (H) protons (∼6.8 and ∼8 ppm) were not observed in the presence of Cu^2+^ indicating restricted molecular motion, which is consistent with the H involvement in the complexation of all of the peptides (Pep1–Pep3, Figure 3B). Importantly, in the spectra of Pep2, we also observed broadening of the lysine (K) signals in the presence of Cu^2+^ (Figure 3B), which is also consistent with reduced mobility due to assembly and complexation. When this amino acid is in the middle but not at the end of the peptide backbone, as in Pep3, the K signals are still present in the NMR spectrum; however, the broadening and downfield shift of the signals in the presence of Cu^2+^ also indicate its participation in complexation.

### 2.2 Stability of the Peptides

The chemical structure and molecular packing of the peptides play a crucial role in their stability by (un)­shielding the amide bonds from enzymatic degradation.^26–29^ We investigated the stability of the supramolecular assemblies generated by Pep1–Pep3 in the presence of proteinase K. This enzyme was selected because of its activity toward a broad range of peptides/proteins that spans different temperatures (25–65 °C) and pH values (7–12).

In the absence of Cu^2+^, all the peptides were susceptible to proteolysis. HPLC analysis showed that proteinase K causes a steady decrease of their concentration over time, reaching a degradation of 70–90% after 24 h (Figure 4).

**Figure 4:**
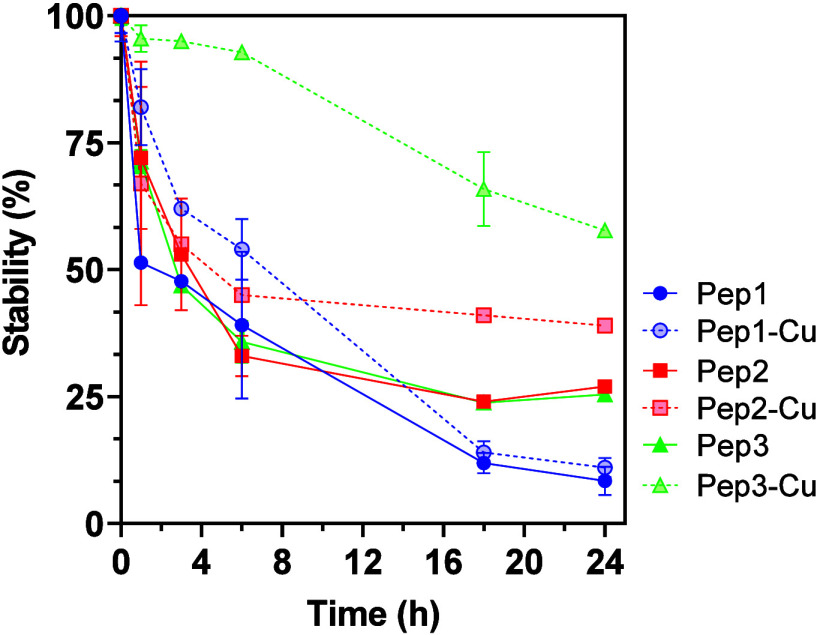
Effect of proteinase K on the stability of the peptides in the absence (Pep1–Pep3) and in the presence of Cu^2+^ (Pep1-Cu, Pep2-Cu, and Pep3-Cu). All peptides were used at a concentration of 2 mM.

The addition of Cu^2+^ increased the stability of all peptides (Figure 4, dashed lines). At these conditions, Pep3 was the most stable; more than 90% were not degraded after 6 h of incubation with proteinase K and 60% of its initial concentration was still present at the end of the studied period (after 24 h). On the other hand, Pep1 was the peptide more susceptible to enzymatic degradation. These results can be explained with the endolytic activity of the proteinase K, i.e., its action requires access to the internal peptide bonds that is hindered by the supramolecular assembly. Additionally, the closer packing promises more effective hindrance, i.e., the effect of Cu^2+^ on the enzymatic degradation can be explained by a denser packing that results from its complexation and inhibiting the activity of proteinase K.

Altogether these data indicated that Pep3 may have high bioactivity because of the enhanced biostability resulting from the self-assembly and the prompt complexation with Cu^2+^ due to the beneficial topology of the amino acids in this heptapeptide.

### 2.3 Cytocompatibility of the Peptides

As the designed systems target wound healing, we assessed their cytocompatibility toward human keratinocyte cell line HaCaT. Keratinocytes are ubiquitous in the skin and often used for *in vitro* assays of new therapeutics for a variety of skin conditions, including wound healing.^30,31^

Upon GHK supplementation, we observed a concentration-dependent increment of HaCaT metabolic activity (Figure S12) that is consistent with the proteolytic instability of the GHK: at lower concentrations, most of the supplemented peptide is degraded and we did not observe the expected increment in activity, but higher concentrations stimulated the cells’ metabolism. On the other hand, HaCaT metabolic activity was significantly higher for cultures supplemented with coassembled Pep1 or conjugated heptapeptides at all the tested concentrations, i.e., between 5 and 1000 μM (Figures S12A and S12B). When the assembly was performed in the presence of Cu^2+^, we observed cytotoxicity at higher concentrations (above 250 μM for Pep2 and Pep3 and above 500 μM for Pep1). A similar cytotoxic effect has been observed previously and was assigned to the presence of free (uncomplexed) Cu ions.^32^ Among the tested peptides, Pep3 has the most notorious effect and enhanced the metabolic activity of HaCaT cells under all studied conditions. Because the highest noncytotoxic concentration was 250 μM (Figure S12C), we selected this concentration to assess the peptide ability to promote wound healing.

### 2.4 Induction of Wound Healing

During skin wound healing, the establishment of a cohesive keratinocytes layer plays a pivotal role in the re-epithelialization process.^1,2^ This process involves activation, migration, and proliferation of epidermal stem cells.^1,3^ Previous data have shown that GHK-Cu can accelerate the wound healing.^7,13–15^ We conducted a scratch wound-healing migration assay and observed that the three peptides enhanced cell migration, but the effect of Pep3 (with or without Cu^2+^) was the most significant (Figure 5A). The re-epithelialization process usually begins 16–24 h postinjury.^1^ The calculated closure rates after 24 h of contact with the peptide assemblies showed an increment in the HaCaT proliferation when compared with the experiments using only GHK or GHK-Cu (Figure 5A). This tendency was more pronounced for Cu-complexed peptides that enhanced the closure rates of 1.7-fold for Pep1-Cu and Pep2-Cu and 2.7-fold for Pep3-Cu. After 24 h, residual scratched wound areas below 50% for Pep2 and Pep3 were measured (Figure 5 and Figure S13). Notably, Pep3-Cu induced a wound closure rate of 73% after 24 h (Figure S13A). Surprisingly, no significant difference was observed between HaCaT cells exposed to Pep1/Pep1-Cu and GHK-Cu, indicating that the coassembly of F_4_D and GHK does not increase GHK bioactivity. A possible explanation for these results is the disruption of the electrostatic interactions between the two peptides by the ions present in the culture medium, leading to the exposure of the GHK tripeptide out of the vicinity of the F_4_D nanoassemblies (Pep1).

**Figure 5:**
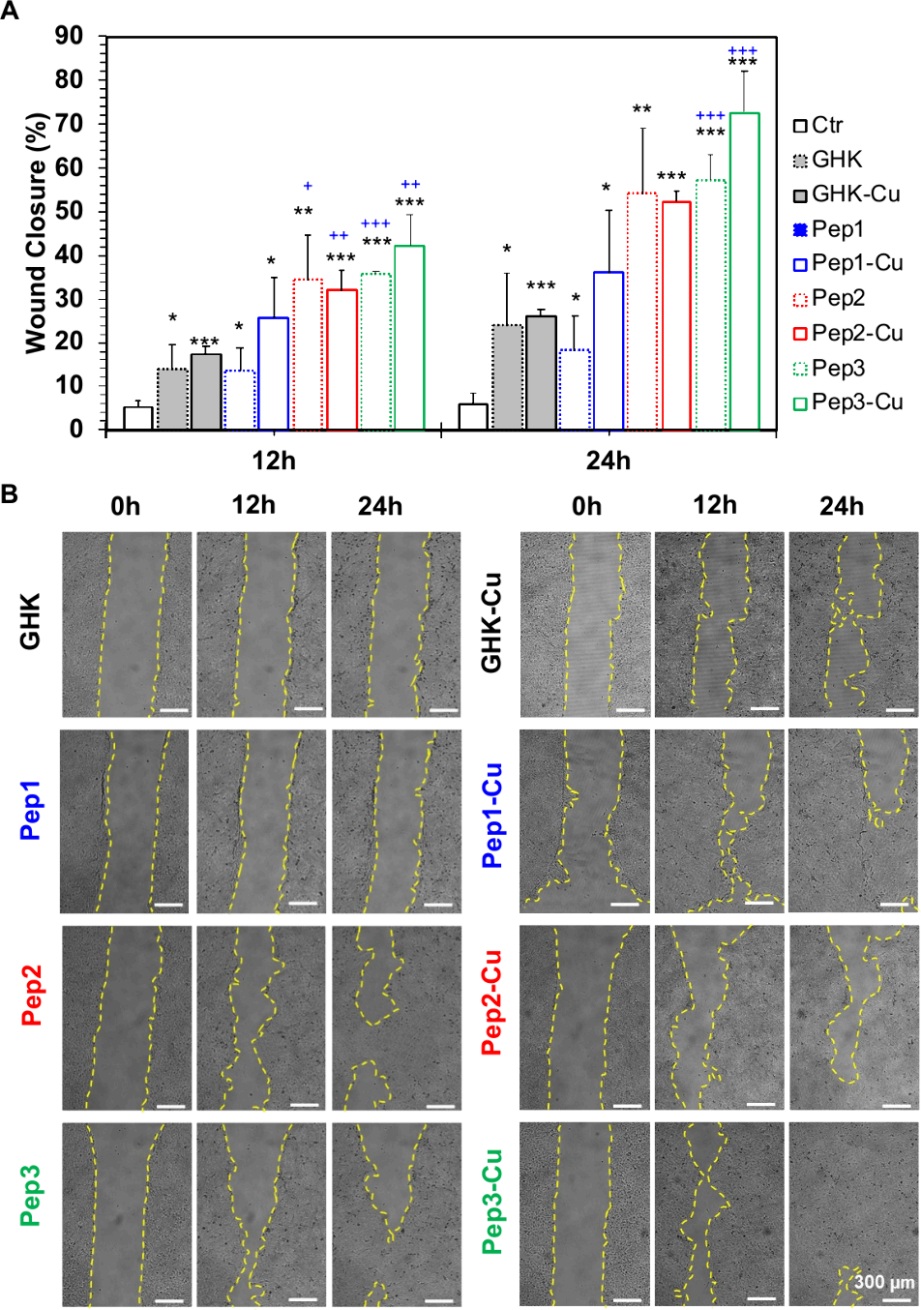
Scratch wound-healing migration assay of HaCaT cells exposed to the studied peptides (250 μM) in the presence and absence of Cu^2+^. (A) Quantitative analysis of wound closure percentage cells after 12 and 24 h of culture. Statistical analysis performed using a comparison to HaCaT cells in serum-free culture medium (*p < 0.05, **p < 0.01, and ***p < 0.001) or HaCaT cells cultured with GHK-Cu (^+^ *p* < 0.05, ^++^ *p* < 0.01, and ^+++^ *p* < 0.001). (B) Representative optical microscopy images of migration of HaCaT cells in the presence of the peptides/Cu^2+^ and at different time points (0, 12, and 24 h). Scale bar = 300 μm.

As mentioned above, the first phase of healing is the inflammation. During this phase, keratinocytes release pro-inflammatory cytokines that promote their migration in the following proliferation/migration phase, i.e., the inflammation acts as a regulatory force that, ultimately, leads to wound healing.^1,33,34^ The timing and duration of the inflammation process are crucial for the healing. As an example, an early release of interleukin-6 (IL-6) triggers a proinflammatory response, while its later release leads to a reparative, anti-inflammatory response contributing to different stages of wound healing.^35^

To assess the peptide activity during the inflammation phase, we exposed HaCaT cells to an inflammatory stimulus induced by interferon γ (IFN-γ) and tumor necrosis factor alpha (TNF-α). We used a multiplex immunoassay (MAGPIX Luminex technology) to evaluate the cellular response by quantifying the expression of IL-6 and IL-8. IL-6 was selected because it promotes keratinocyte migration and proliferation and its deficiency reduces neutrophil and macrophage infiltration while inhibiting keratinocyte proliferation.^3,33,35^ IL-8 was chosen because it is a key mediator of inflammation linked to keratinocyte migration. It acts as a chemoattractant of proinflammatory cells, playing a crucial role in facilitating proper damage repair.^31,32,36^ Our data showed that the covalent peptides Pep2 and Pep3 induced an increased expression of these proinflammatory cytokines at the initial stage (3 to 6 h, consistent with the inflammation stage of the wound healing process), indicating their efficacy in the immunogenic inflammation process (Figures 6A–6D). In the next proliferation/migration phase (i.e., ∼16–18 h after damage), the peptides continued to induce expression of IL-6 and IL-8 (Figures 6E and 6F) with a significant effect of Pep3-Cu in comparison with the other tested conditions (Figures 6G and 6H). This result is consistent with the proteolytic degradation data showing that Pep3-Cu is the most stable (Figure 4).

**Figure 6:**
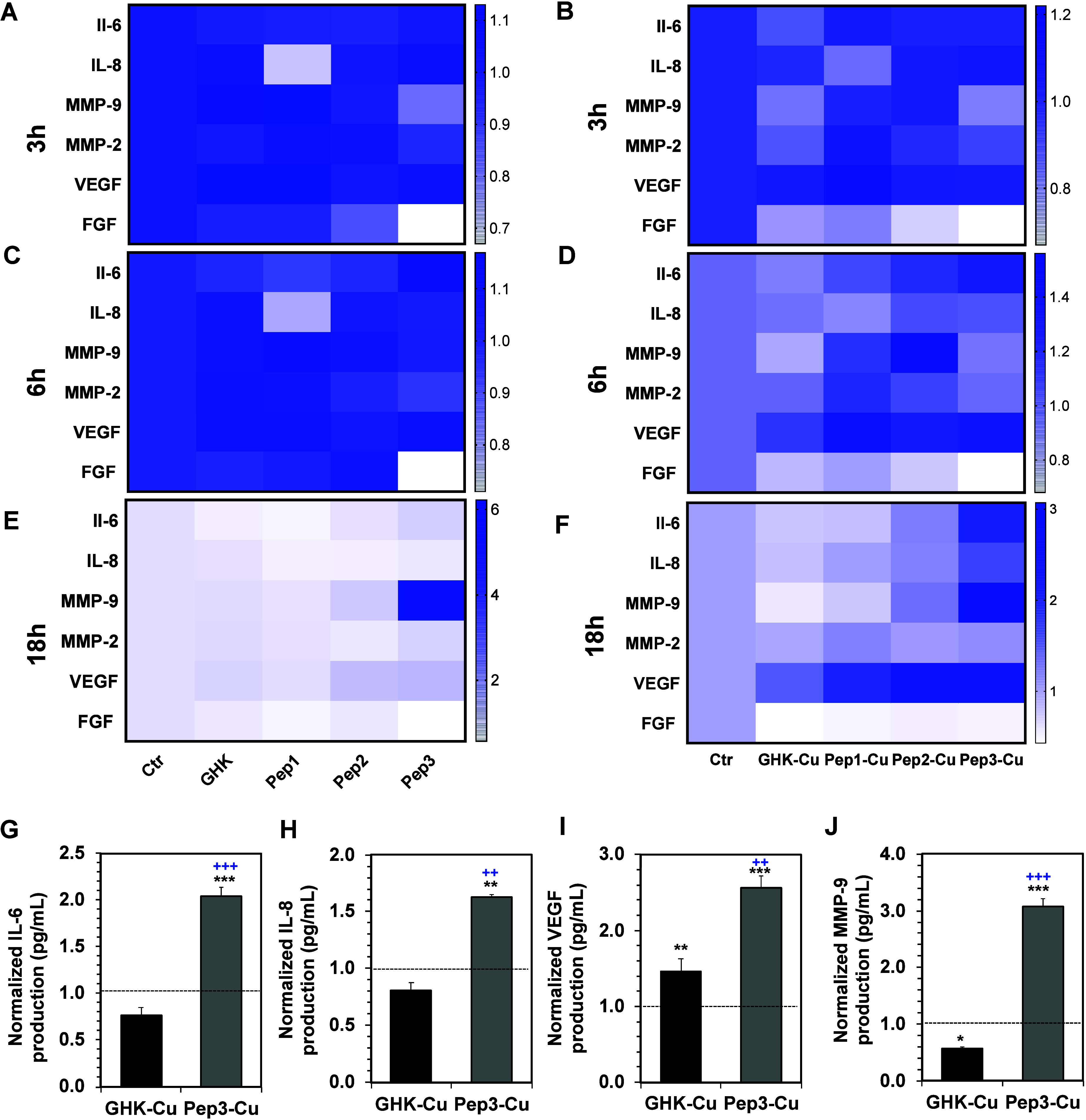
(A–F) Heatmaps showing the expression of wound healing markers (IL-6, IL-8, MMP-9, MMP-2, VEGF, and FGF) at (A and B) 3 h, (C and D) 6 h, and (E and F) 18 h of HaCaT cell cultures supplemented with (A, C, and E) the different peptides (Pep1–Pep3) or (B, D, and F) their complexes with Cu^2+^ (Pep1-Cu, Pep2-Cu, and Pep3-Cu). All values were normalized to the control. (G–J) Quantification of the expression of selected markers by HaCaT cultures (at 18 h of cell culture) supplemented with GHK-Cu and Pep3-Cu. Statistically significant differences marked as *p < 0.05, **p < 0.01, and ***p < 0.001 (compared to control, i.e., cells in the absence of peptides) and ^++^ *p* < 0.01 and ^+++^ *p* < 0.001 (compared to GHK-Cu).

We also assessed the expression of matrix metalloproteinases (MMPs). Once inflammation is controlled, re-epithelialization follows, for which MMPs are essential to disrupt the basement membrane and allow keratinocyte migration through the wound matrix.^1,37^ Additionally, MMPs are involved in regulating angiogenesis by activating proangiogenic cytokines (e.g., TNF-α and vascular endothelial growth factor, VEGF)^37^ needed for recruitment of endothelial cells for capillary formation.^1^ Our results showed an overexpression of MMP-9 at the 18 h time point (Figures 6E, 6F and 6J). As in the case of cytokines, the effect of Pep3-Cu on the MMP-9 expression was most significant and consistent with the observed increment in keratinocyte migration and proliferation (Figures S12 and S13).

Overexpression of VEGF and bFGF is prominent in the proliferative phase of wound healing, as these factors effectively enhance vascularization and re-epithelialization.^1,33^ Previous studies have shown that GHK-loaded alginate hydrogels promote the secretion of VEGF and bFGF by mesenchymal stem cells and human umbilical vein endothelial cells, thereby increasing cell proliferation and differentiation.^14,15,38,39^ Pep1–Pep3 did not increase the expression of bFGF but induced an overexpression of the angiogenic VEGF by HaCaT cells (Figures 6A–6F), which is a critical factor in wound healing. In line with the results obtained for the quantification of the pro-inflammatory cytokines and metalloproteinases, the highest increment in VEGF secretion was observed in the presence of Pep3-Cu (Figure 6I).

Altogether the data confirmed that the conjugation of the tripeptide GHK/KHG to the F_4_D is a successful strategy to enhance the bioactivity of the GHK sequence by reducing its enzymatic degradation. The increased bioactivity may also be derived from increased Cu^2+^ retention in the nanofibers/nanotapes.

### 2.5 Preparation and Biological Assessment of a Hydrogel Patch

Because of the prominent results obtained with Pep3-Cu, we explored the possibility of developing a hydrogel patch from this peptide targeting skin wound healing. Hydrogels are known for their efficacy in skin regeneration due to their structural resemblance with the extracellular matrix, a hydrated fibrillar network.^40–42^ Moreover, peptide-based hydrogels offer advantages as specific bioactivity can be coded by the used sequences and the degradation products, amino acids, are not cytotoxic.^40,41^

At higher concentration (30 mM) Pep3-Cu forms a viscous solution that upon contact with the ions from the cell culture medium formed hydrogel (Figures 7A and 7B). Rheological characterization of the obtained hydrogels revealed an elastic modulus (*G*′) of approximately 130 kPa (Figure S14), which is in the range of the skin modulus, i.e., between 50 kPa (for the dermis) and 4 MPa (for the epidermis).^43^ The sol–gel transition can alter material cytotoxicity. In the case of Pep3-Cu, we observed reduced cytotoxicity of the hydrogel in comparison to that of the free nanofibers/nanotapes in suspension: HaCaT cells were cultured on the surface of Pep3-Cu hydrogel and, after 24 h, a live/dead assay showed a monolayer of live cells (Figure 7C).

**Figure 7:**
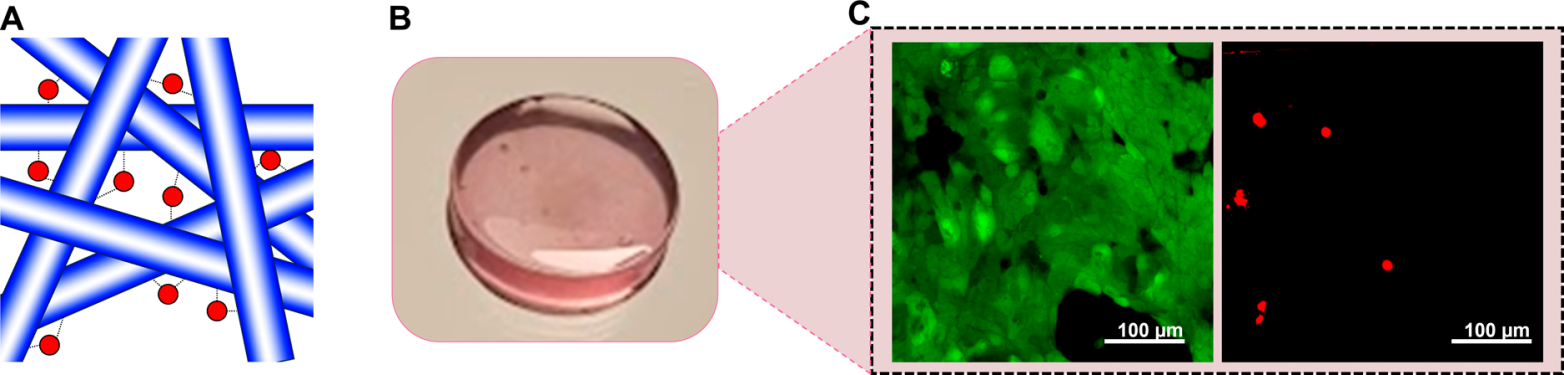
(A) Scheme showing the cross-linking of the nanofibers/nanotapes by the ions of the medium (red dots) leading to the formation of the hydrogel matrix. (B) Macroscopic appearance of the Pep3-Cu hydrogel (concentration 30 mM). (C) Confocal microscopy images of HaCaT cells cultured for 24 h on Pep3-Cu hydrogel stained with calcein AM (left, green, live cells) and ethidium homodimer-1 (right, red, dead cells). Scale bars = 100 μm.

The possibility of generating supramolecular hydrogels from bioactive peptides has several advantages over common delivery strategies using vehicles as liposomes or standard hydrogels: it avoids the use of cross-linking agents often applied for the preparation of conventional hydrogels and does not depend on loading efficiency (a challenge associated with the use of liposomes as delivery vehicles) as the bioactive peptide itself is the building block of the hydrogel.^21^ Moreover, this possibility demonstrates that Pep3-Cu is a versatile system that can be used under different administration concepts, i.e., in the form of a suspension or as a hydrogel.

## 3 Conclusions

We successfully applied nanoprotein engineering to enhance the bioactivity of GHK. We found that conjugation of the structural sequence (F_4_D) at the lysine of KHG (inverted sequence of GHK) forming F_4_D-KHG (Pep3) presents higher stability to proteolytic degradation than the GHK-based counterparts. Moreover, this peptide has higher bioactivity than GHK, and it forms a complex with Cu^2+^ that promotes more efficient wound closure (tested using scratch wound assay), reaching a closure percentage of approximately 70% in 24 h. Finally, we provide a proof of concept for the application of this peptide as a hydrogel patch for skin wound healing.

## 4 Experimental Section

### 4.1 Synthesis and Purification of Peptides

The peptides were synthesized via solid-phase peptide synthesis (SPPS) in an automated peptide synthesizer (CS Bio) applying standard 9-fluorenylmethoxycarbonyl (Fmoc) protection chemistry. Wang resin preloaded with the first amino acid was utilized (Fmoc-Lys­(Boc)-Wang resin or Fmoc-Asp­(OtBu)-Wang, Novabiochem). All following couplings involved 4 mmol equivalents of Fmoc-protected amino acids (Fmoc-His­(Trt)-OH, Fmoc-Gly-OH, Fmoc-Phe-OH, and Fmoc-Asp­(OtBu)-OH purchased from Novabiochem and Fmoc-Lys­(Boc)-OH from Sigma-Aldrich), 4 mmol equivalents of 2-(1H-benzotriazole-1-yl)-1,1,3,3-tetramethyluronium hexafluorophosphate (HBTU, Novabiochem), and 6 mmol equivalents of *N*,*N*-diisopropylethylamine (DIEA) (Sigma-Aldrich) in *N*,*N*-dimethylformamide (DMF, Honeywell Riedel-de Haën) for 1 h. Fmoc deprotection was performed with 20% piperidine (Sigma-Aldrich) in DMF, for 20 min.

Peptide cleavage from the resin and side chain deprotection was carried out by a cocktail of trifluoroacetic acid, triisopropylsilane, and water (TFA:TIS:water) at a ratio 95:2.5:2.5 (TFA and TIS were purchased from ThermoFisher) for 3 h at room temperature. After filtration, the resin was washed with dichloromethane (DCM, Honeywell Riedel-de Haën). DCM and TFA were removed by evaporation, and the crude product was precipitated using cold diethyl ether (Honeywell Riedel-de Haën). The precipitate was collected by filtration, washed with cold diethyl ether, dissolved in water, and freeze-dried. Purification was achieved using a preparative high-performance liquid chromatography (HPLC) equipped with a Waters 2545 Binary Gradient Module liquid chromatography system (Waters Corporation) and an Atlantis preparative T3 C18 column (5 μm, 30 × 250 mm, Waters Corporation). A gradient of water (A) and acetonitrile (B) (ACN, Riedel-de Haën) with 0.1% TFA was used as a mobile phase (Table S1), and a flow rate of 10 mL/min, a loop of 5 mL, and detection wavelength at 220 nm was used for the purification. To remove the residual TFA, fractions were evaporated, and the solid was dissolved in 100 mM HCl (Riedel-de Haën). The solution was freeze-dried to obtain the peptide hydrochloride salt. Their purity was assessed by analytical HPLC (Alliance, Waters Corporation) using a C18 Atlantis column (250 mm × 4.6 mm, 5 μm, Waters Corporation). The peptide structure was confirmed by mass spectroscopy (Quattro Micro API, Waters Corporation) using an electrospray ionization mass spectrometer (ESI-MS) in positive-ion mode and nuclear magnetic resonance spectroscopy (NMR, 400.13 MHz Avance III spectrometer, Bruker) in DMSO-*d*_6_ or D_2_O (also used as an internal reference for chemical shifts δ, which are reported in ppm).

### 4.2 Formation of the Supramolecular Structures and Hydrogels

Supramolecular assemblies of Pep1 were prepared by suspending F_4_D (20 mM) and GHK (20 mM) in phosphate-buffered saline (PBS), followed by addition of 2 M NaOH until pH ≈ 10 (for the F_4_D solution). Mixing of F_4_D and GHK solutions in a 1:1 ratio resulted in a final concentration of 10 mM of Pep1. The pH of this solution was adjusted to ∼7.5 using 0.1 M HCl to trigger sol–gel transition. For the Cu-complexation experiments, a 0.6 M CuCl_2_ aqueous solution was added to Pep1 before the pH was adjusted to 7.5. The color change (white to blue) served as a confirmation of the presence of Cu^2+^ (Figure S15).

In the case of Pep2 or Pep3 (10 mM), they were suspended in PBS, and the pH was adjusted by dropwise addition of 2 M NaOH until complete dissolution of the peptides (pH ≈ 10). Complexation with Cu^2+^ was performed by the addition of a 0.6 M CuCl_2_ aqueous solution. The assembly was then triggered by adjusting the pH to ∼7.5 (using 0.1 M HCl) in both Cu-free and Cu-complexation studies.

Hydrogels of Pep3 were prepared using the same method, but the concentration was increased to 30 mM. A pregel solution (100 μL) was added into 24-well ThinCert cell culture inserts (Greiner Bio-One, GmbH). These inserts, containing the pregelation solution, were placed into 24-well culture plates with 1 mL of culture medium in each well. The plates were incubated for 3 h at 37 °C and 5% CO_2_, followed by medium replacement and the addition of 100 μL of medium on the top of the gels.

### 4.3 ^1^H Nuclear Magnetic Resonance Spectroscopy

^1^H NMR spectra of peptide assemblies (free or complexed) were recorded on a Bruker 400.13 MHz Avance III spectrometer. Peptides and Cu powders were solubilized in D_2_O and prepared by using the method described in Section 4.2 to form the supramolecular structures.

### 4.4 Circular Dichroism (CD) Spectroscopy

The secondary structure of peptides was analyzed using a Jasco J1500 spectrophotometer (Jasco Corporation) with a 0.2 cm quartz cell. Peptide solutions (10 mM) were prepared 24 h in advance and diluted to 2 mM immediately before the CD analysis. Spectra were recorded at a scan speed of 200 nm·min^–1^, a data pitch of 0.5 nm, and a bandwidth of 1 nm. Three acquisitions were accumulated for each sample.

### 4.5 Atomic Force Microscopy (AFM)

Peptide assemblies were prepared 24 h in advance and diluted in water. A droplet of the diluted peptide sample was carefully deposited on a mica sheet and subsequently dried under a gentle nitrogen flow. The mica surface was rinsed with water and again dried using nitrogen. AFM images were acquired using a JPK Nanowizard 3 (JPK) in air at room temperature under AC mode. The scans were acquired at a 512 × 512 pixel resolution using ACTA-SS probes (*k* ≈ 37 N/m, AppNano, Scientec), a drive frequency of ∼254 kHz, a set point of ∼0.5 V, and a scanning speed of 1.0 Hz. Images were analyzed by using the JPK Data Processing software.

### 4.6 Rheological Characterization of the Pep3-Cu Hydrogels

The mechanical performance of the hydrogels generated by Pep3-Cu were characterized using a strain-controlled rheometer (Kinexus Pro, Malvern) equipped with an 8 mm parallel plate geometry. Briefly, hydrogels were prepared as described in Section 4.2. One day prior to the assay, hydrogels were cut using a puncher (with a diameter of 8 mm), positioned on the rheometer’s plate, and the gap was adjusted. Experiments were carried out at a constant temperature of 37 °C. The linear viscoelastic region (LVER) was determined by conducting amplitude sweep measurements at a frequency of 10 Hz with strain values ranging from 0.1% to 10%. Using the LVER data, the elastic modulus (*G*′) and viscous modulus (*G*″) were measured at a frequency of 10 Hz and a strain of 0.05%. All measurements were performed in triplicate, and average values were calculated.

### 4.7 Stability of Peptides against Proteinase K

A stock solution of proteinase K (50 μg/mL, AppliChem) was prepared in PBS. Peptide assemblies were prepared as described in Section 4.2 1 day prior the assay. These samples were incubated with proteinase K (final concentration: 10 μg/mL for proteinase K and 2 mM for Cu-free or Cu-complexed Pep1-Pep3). At each designated time point, an aliquot of 150 μL was collected, and the enzymatic activity was stopped by adding 300 μL of 1:1 H_2_O/ACN with 0.1% TFA. The aliquot was quantitatively assessed using HPLC under the conditions described in Section 4.1. Each sample was run in duplicate to ensure reliable results.

### 4.8 Cell Culture

Immortalized human keratinocytes (HaCaT cells) were used for cellular assays. HaCaT cells were cultured in high glucose Dulbecco’s modified Eagle’s medium (DMEM-HG, Sigma-Aldrich) supplemented with 10% fetal bovine serum (FBS, Gibco) and 1% antibiotic solution (AT; final concentration of penicillin 100 units/mL, streptomycin 100 mg/mL and 25 μg/mL amphotericin B; Gibco) and were maintained in a humidified atmosphere at 37 °C and 5% CO_2_ until confluence. Culture media were changed every other day.

### 4.9 Cytotoxicity of Supramolecular Peptides

The cytotoxicity evaluation of the peptides and their supramolecular assemblies was evaluated by direct contact assay with HaCaT cells using the AlamarBlue HS Cell Viability Reagent assay (Biorad). Briefly, HaCaT cells were cultured in DMEM-HG supplemented with 10% FBS (2.5 × 10^4^ cell/well) in 96-well plates for 24 h. Peptide solutions (10 mM) were prepared 24 h before the assay and sterilized (UV, 30 min). These solutions were diluted in serum-free DMEM-HG just prior to the assay to obtain concentrations ranging from 1 to 0.05 mM and added to the cultured HaCaT cells. Cultures with the peptides were mantained for 24 h and then the medium was aspirated, and AlamarBlue HS Cell Viability solution (10% (v/v) in DMEM-HG) was added to each well. The plates were incubated for 3 h at 37 °C with 5% CO_2_. The medium from each well was transferred to a black 96-well plate, and the fluorescence was measured using a microplate reader (Synergy, Bio-Tek) with an excitation wavelength of 550 nm and an emission wavelength of 590 nm. Cells cultured on tissue culture polystyrene (TCPS) were used as a positive control and cells treated with 20% DMSO were used as a negative one.

### 4.10 Cell Viability

The cellular viability was evaluated by a live/dead assay. Briefly, HaCaT cells were seeded at a density of 1.5 × 10^5^ cells per well in 24-well plates and cultured in DMEM-HG supplemented with 10% FBS for 24 h. Peptide assemblies (250 μM, either free or Cu-complexed) in DMEM-free medium were added to each well, and after 24 h of culture, the medium was removed and cells were incubated for 10 min at 37 °C in a staining solution consisting of Calcein-AM (2:1000, Invitrogen) and ethidium homodimer-1 (EthD-1, 1:1000, Sigma-Aldrich) in PBS. Cellular observations were made using an inverted microscope with thunder (DMi8, Leica). For cells cultured on the hydrogel surface, HaCaT cells were incubated for 20 min with the staining solution, followed by a wash with PBS. Gels were then removed from the insets and observed under a confocal laser scanning microscope (TCS SP8, Leica).

### 4.11 Wound-Healing Scratch Assay

A scratch wound assay was performed in a Culture-Insert 2 Well in a μ-Dish 35 mm (Ibidi). HaCaT cell suspension (70 μL, density of 6 × 10^5^ cells per mL) in serum containing DMEM-HG was added in each chamber and cultured for 24 h. After removing the culture inserts and washing the unattached HaCaT with serum-free medium, 250 μM peptide assemblies (Cu-free or Cu-complexed) were added into each well, and the attached cells were cultured in an inverted microscope with incubation (Axio Observer, Zeiss) for 24 h at 37 °C with 5% CO_2_. Phase-contrast images were captured using the same microscope, and the closure area was calculated using the following formula:

$$\mathrm{migration area}\;\left(\%\right)=\left(A_{0}-A_{n}\right)/A_{0}\times 100$$

where *A*_0_ represents the initial wound area and *A*_n_ is the remaining area of the wound at the measurement point. The closure rate of the wound was determined by the following formula:

$$\mathrm{closure rate}\;\left({\mu m}^{2}/h\right)=\left(A_{0}-A_{n}\right)/t$$

where *t* is the time of each data point in h.

Quantitative analysis of migrated cells was performed using ImageJ (version 1.53t, plug-plot *Z*-axis Profile).

### 4.12 Cytokine Assay

The inflammation in HaCaT cells was induced by the addition of TNF-α (cat. no. 300-01A; PeproTech) and IFN-γ (cat. no. 300-02; PeproTech). Briefly, HaCaT cells were cultured in a 24-well plate at a density of 1.5 × 10^5^ cell per well in serum-containing DMEM-HG medium. After 24 h, the medium was replaced with serum-free DMEM-HG supplemented with TNF-α/IFN-γ (10 ng/mL each) and incubated for an additional 3 h. Afterward, peptide assemblies (free or Cu-complexed), at a concentration of 250 μM in serum-free DMEM-HG, were administrated. The cell culture medium was collected at 3, 6, and 18 h and frozen at −80 °C. To quantify the secreted cytokines, MAGPIX Luminex technology (MAGPX20114721, Luminex Corporation) was used following the Human Magnetic Luminex Assays (LXSAHM-10, R&D Systems) protocol. Analyte concentrations were determined by fluorescence using a standard reference curve. xPONENT software (Luminex Corporation, USA) was employed for data analysis.

### 4.13 Statistical Analysis

The results are expressed as the mean ± standard deviation. The normality of the data was assessed using the Shapiro–Wilk test (*p* < 0.05). All data sets exhibited a normal distribution, and a *t* test was subsequently used to assess significant differences, marked by */^+^ for *p* < 0.05, **/^++^ for *p* < 0.01, and ***/^+++^ for *p* < 0.001.

## Supplementary Material



## Notes

Chemical characterization data of all the synthesized peptides including ^1^H NMR and ESI-MS spectra, as well as HPLC chromatograms; additional characterization of the nanostructures generated by the self-assembling of the peptides, including AFM images and assessment of the cytocompatibility; and complementary results from the scratch wound-healing migration assay and rheological characterization of the supramolecular hydrogels (PDF)
