# Supplementary material for: Nanoengineered Self-Assembling Peptides with Increased Proteolytic Stability Promote Wound Healing
Source: ACS Appl Mater Interfaces. 2025 Feb 12;17(8):11624–33. doi: 10.1021/acsami.4c18221 (PMC12818720; doi:10.1021/acsami.4c18221)
Supplement: Supplementary file 1 [file am4c18221_si_001.pdf]

SUPPORTING INFORMATION

Nanoengineered self-assembling peptides with increased proteolytic stability promote wound healing

Vânia I. B. Castro,[a,b] Ana Rita Araujo, [a,b] Rui L. Reis,[a,b] Iva Pashkuleva,[a,b*] Ricardo A. Pires[a,b*]

[a] 3B's Research Group, I3Bs – Research Institute on Biomaterials, Biodegradables and Biomimetics, University of Minho, Headquarters of the European Institute of Excellence on Tissue Engineering and Regenerative Medicine, 4805-017 Barco, Portugal

[b] ICVS/3B's–PT Government Associate Laboratory, 4805-017 Braga/Guimarães, Portugal

* E-mails: pashkuleva@i3bs.uminho.pt; rpires@i3bs.uminho.pt

Table of contents

S1. Characterization of the synthesized peptides**..3**

1. ^{1}H Nuclear Magnetic Resonance spectroscopy (^{1}H NMR)..3
2. Electrospray Ionisation Mass Spectrometry (ESI-MS)..7
3. High-performance liquid chromatography (HPLC)..10

S2. Characterization of the assembled peptides**...11**

S1. Characterization of the synthesized peptides

1. ^{1}H Nuclear Magnetic Resonance spectroscopy (^{1}H NMR)

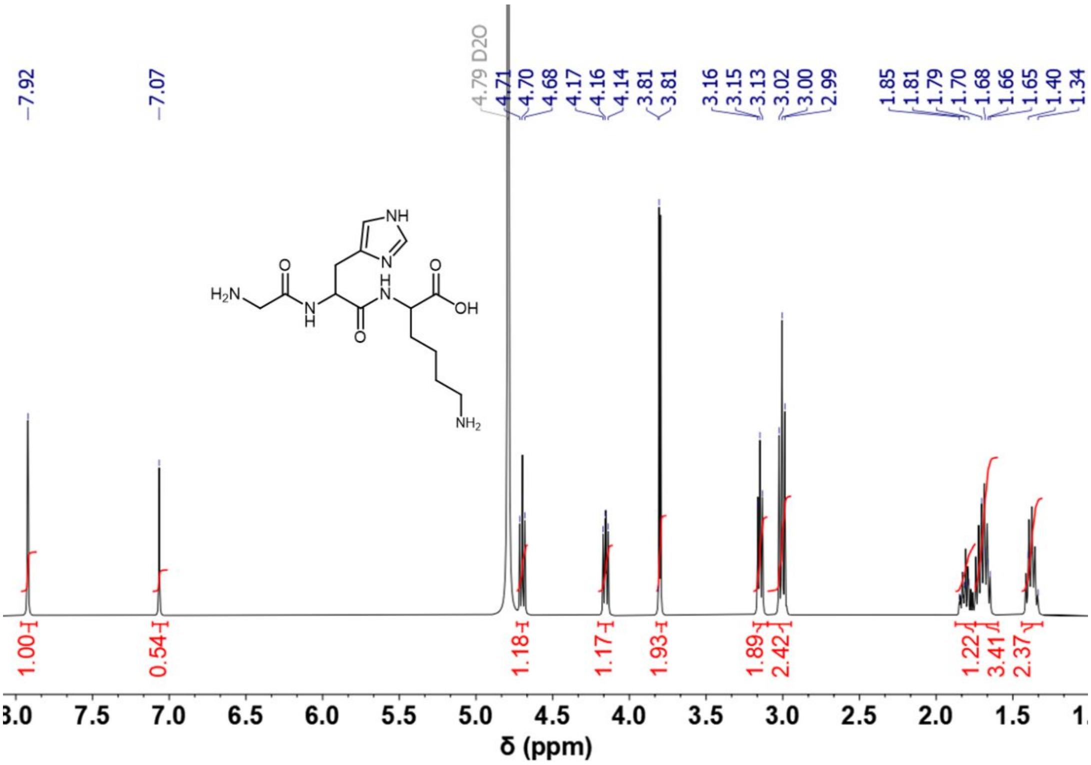

Figure S1. ^{1}H NMR spectrum of the GHK in D_2O at 400 MHz, δ 7.92 (s, 1H), 7.07 (s, 1H), 4.70 (dd, J= 7.2, 6.4 Hz, 1H), 4.16 (dd, J= 7.8, 5.4 Hz, 1H), 3.81 (d, J=4.8Hz, 2H), 3.15, (t, J=6.4Hz, 2H), 3.01 (t, J=7.6Hz, 2H), 1.86-1.77 (m, 1H), 1.75-1.63 (m, 3H), 1.43-1.32 (m, 2H).

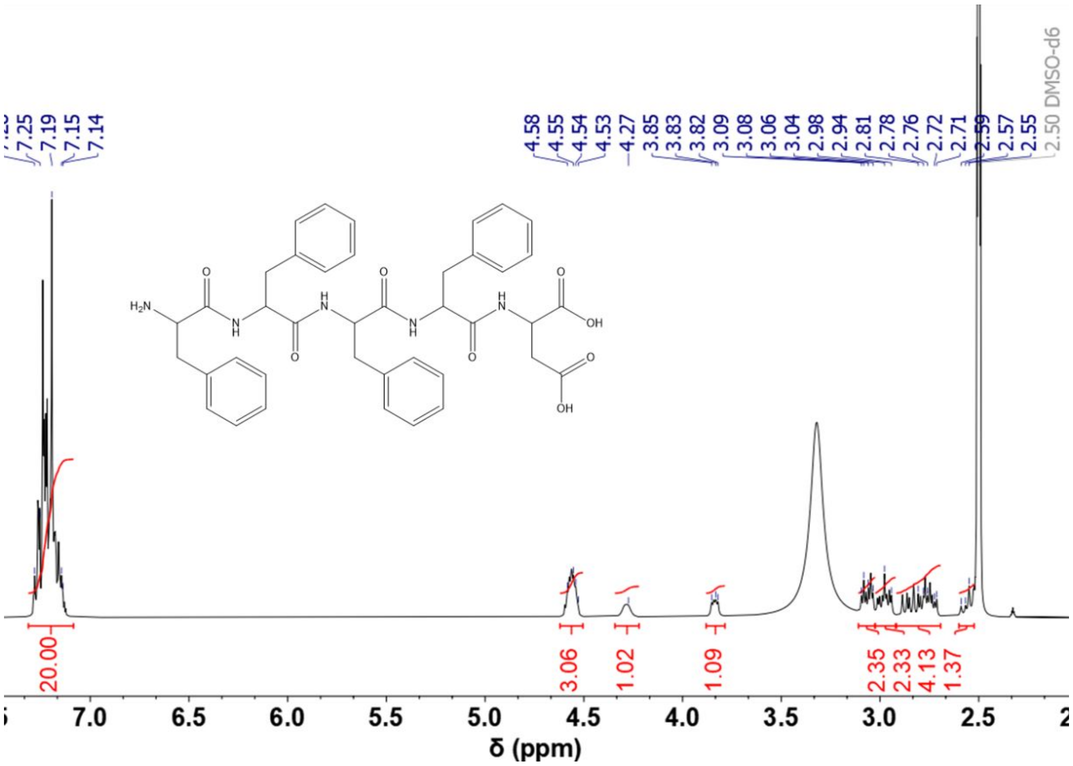

Figure S2. ^{1}H NMR spectrum of F_4D in DMSO at 400 MHz, δ 7.28-7.13 (m, 20H), 4.57-4.52 (m, 3H), 4.31-4.23 (m, 1H), 3.7-3.6 (m,1H), 3.10-3.04 (m, 2H), 2.90-2.72 (m, 4H), 2.60-2.54 (m, 2H).

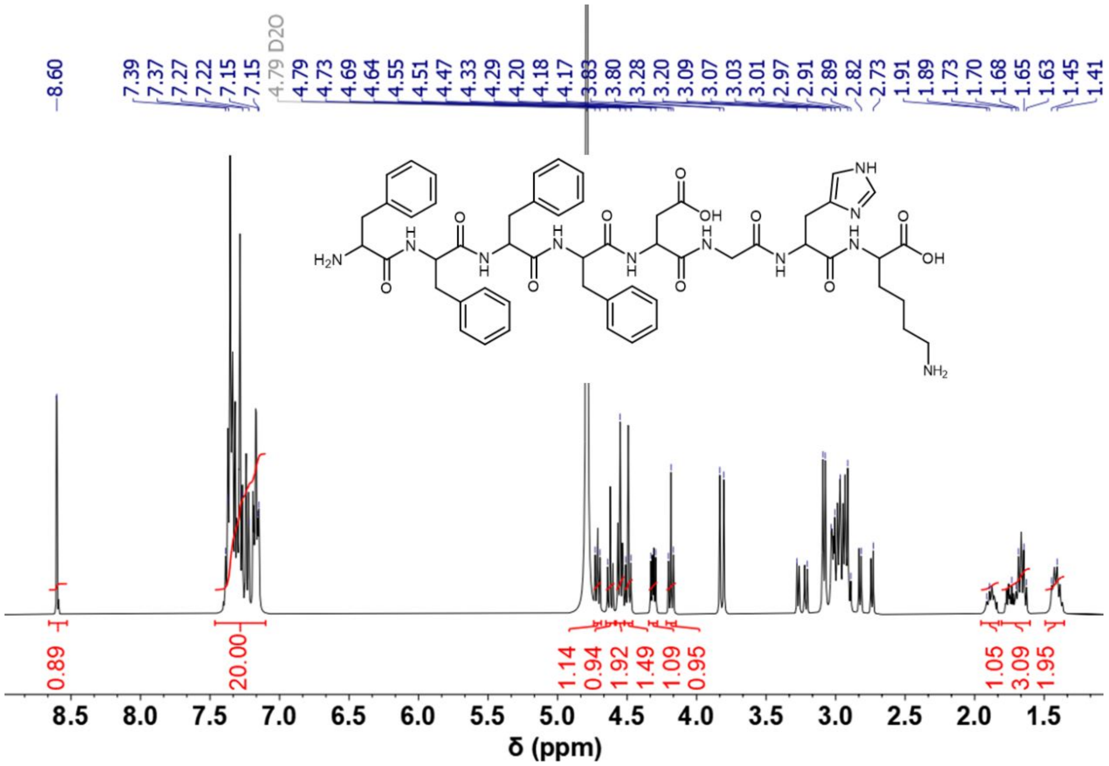

Figure S3. ^{1}H NMR spectrum of F_4D-GHK (Pep2) in DMSO at 400 MHz, δ 8.61 (s, 1H), 7.40-7.14 (m, 21H), 4.71 (dd, J=7.4, 6.3 Hz, 1H), 4.62 (t, J=7.6 Hz, 1H), 4.55, (t, J=6.6 HZ, 2H), 4.49 (t, J=7.6 Hz, 1H), 3.82 (d, J=17.2 Hz, 2H), 3.26 (d, J=6.0 Hz, 1H), 3.20 (d, J=7.6 Hz, 1H), 3.08 (d, J=7.2 Hz, 2H), 3.05-2.86 (m, 8H), 2.82 (d, J=6.4 Hz, 1H), 2.73 (d, J=6.4 Hz, 1H), 1.97-1.83 (m, 1H), 1.77-1.62 (m, 3H) 1.45-1.36 (m, 2H).

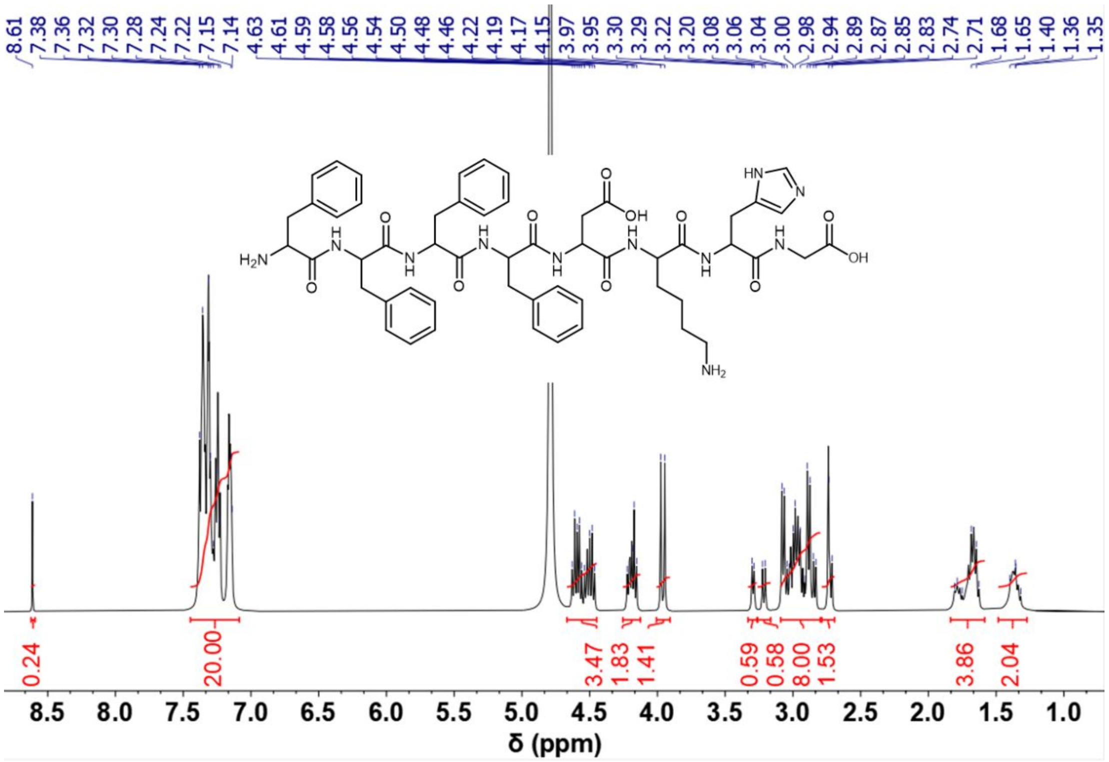

Figure S4. ^{1}H NMR spectrum of F_4D-KHG (Pep3) in DMSO at 400 MHz, δ 8.61 (s, 1H), 7.38-7.22 (m, 17H), 7.18-7.13 (m, 4H), 4.63-4.54 (m, 3H), 4.54-4.45 (m, 2H), 4.23-414 (m, 2H), 3.95 (d, J=11.6 Hz, 2H), 3.29 (d, J=6.0 Hz, 1H), 3.21 (d, J=8.0 Hz, 1H), 3.07 (d, J=7.2 Hz, 2H), 3.05-2.82 (m, 6H), 2.75-2.70 (m, 2H), 1.81-1.74 (m, 1H), 1.73-1.61 (m, 3H), 1.39-1.31 (m, 2H).

2. Electrospray Ionisation Mass Spectrometry (ESI-MS)

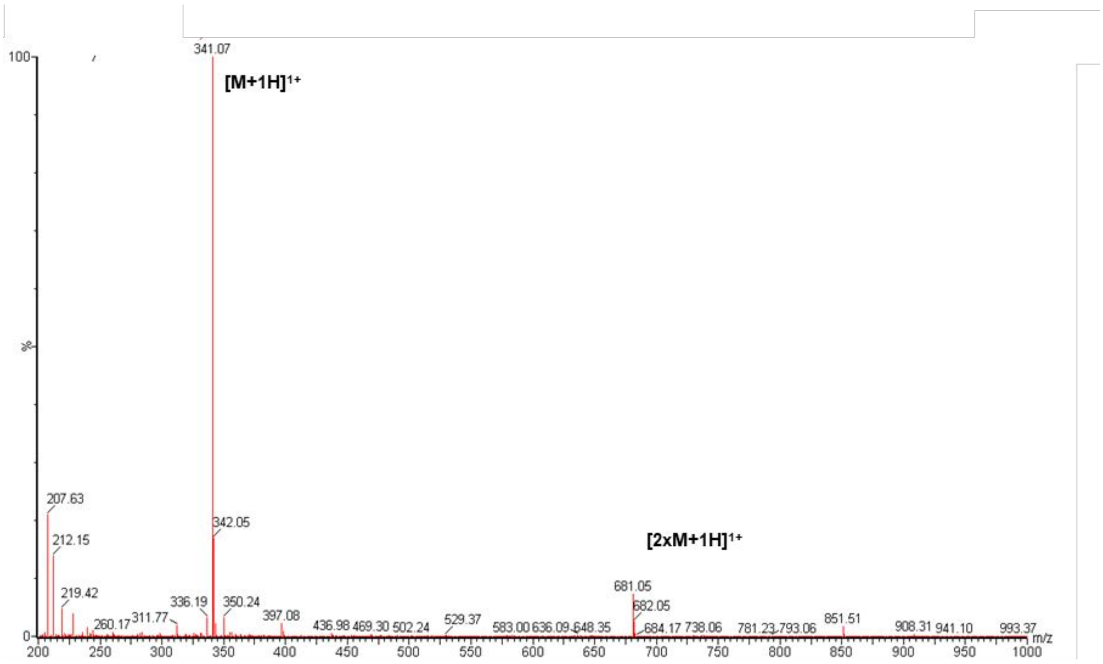

Figure S5. ESI-MS spectrum of GHK (Mw = 340.08g/mol) acquired under positive ion mode.

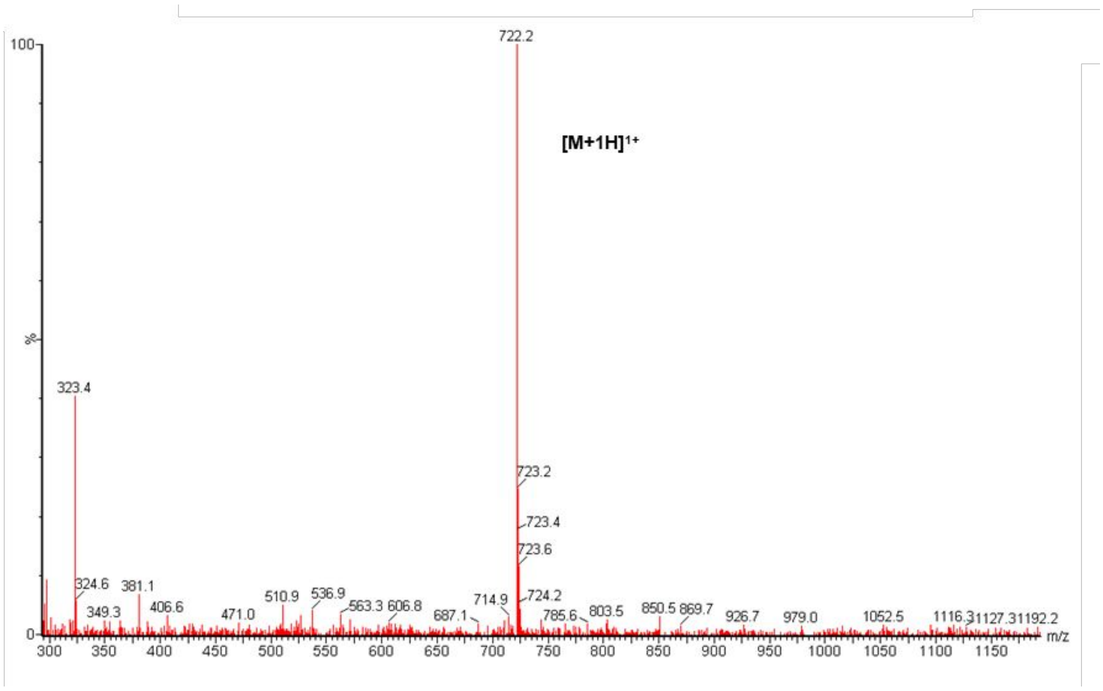

Figure S6. ESI-*MS* spectrum of F_4D (*Mw =* 721.80*g/mol*) acquired *under* positive ion mode.

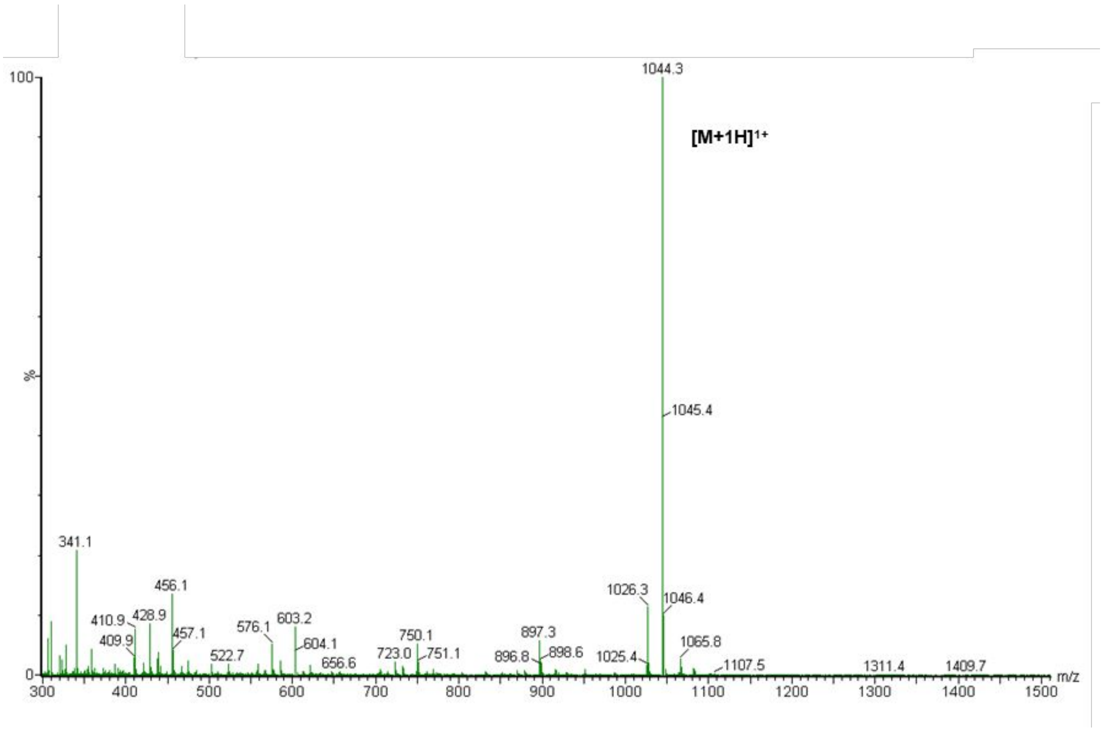

Figure S7. ESI-MS spectrum of F_4D-GHK (Pep2, Mw = 1044.16g/mol) aquired under positive ion mode.

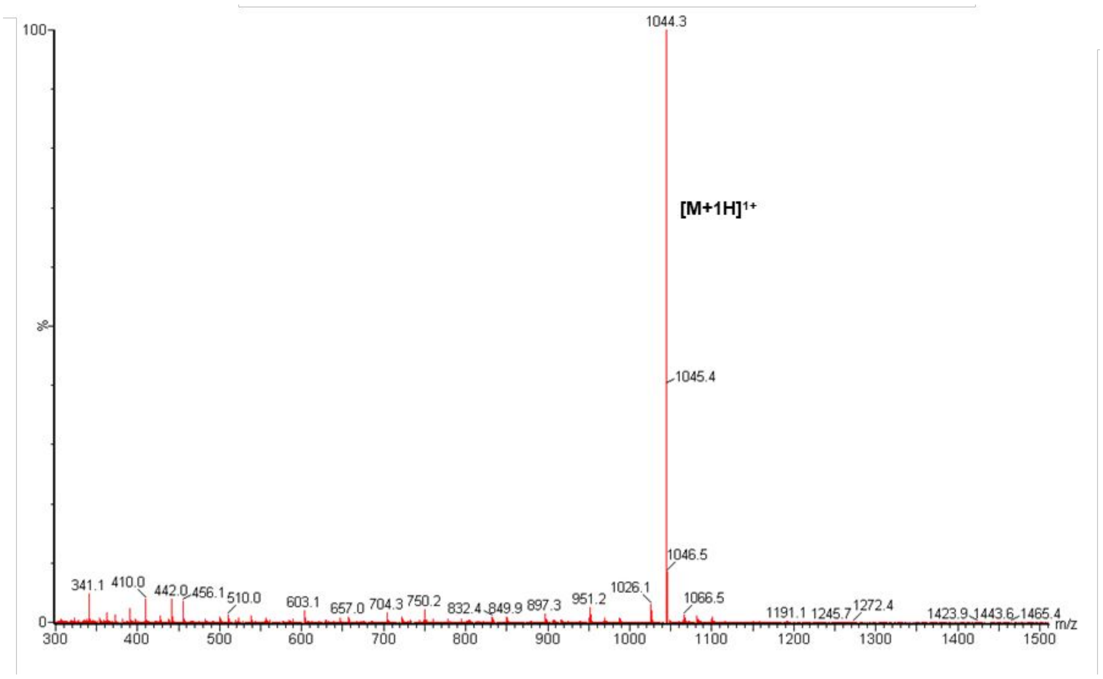

Figure S8. ESI-MS spectrum of F_4D-KHG (Pep3, Mw = 1044.16g/mol) acquired under positive ion mode.

3. High-performance liquid chromatography (HPLC)

Table S1. Mobile phase gradients used during the HPLC runs for peptide characterization as well as for the quantification of peptide stability during the enzymatic degradation assays

	Eluent	
Time (min)	Water + 0.1% TFA	ACN + 0.1% TFA
0	90	10
20	50	50
25	10	90
30	90	10
35	90	10

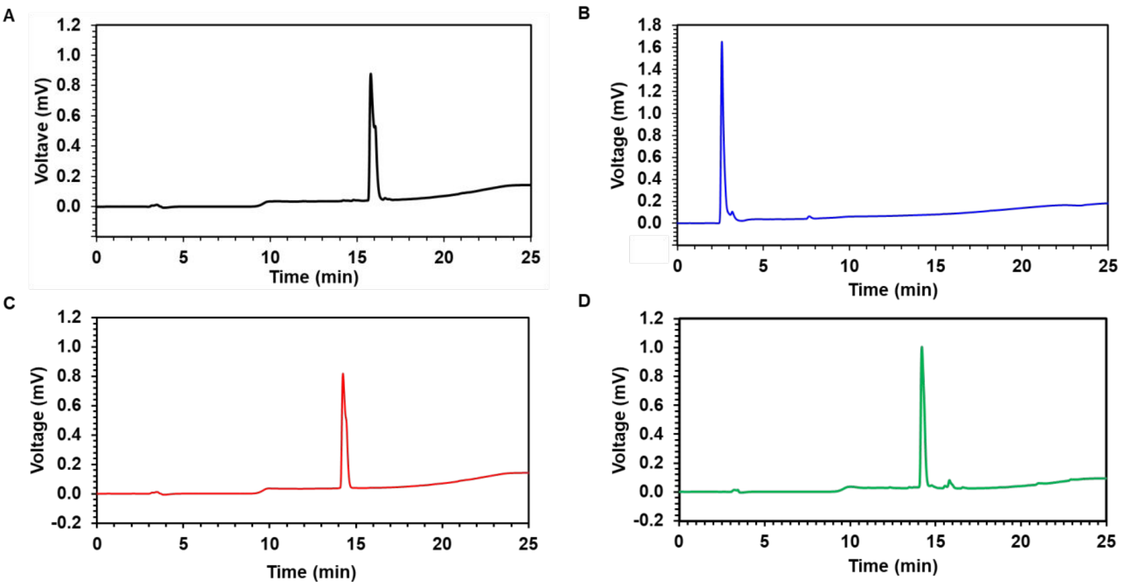

Figure S9. HPLC of the synthesised peptides. **(A)** F_4D (98.3% pure, retention time (tr) = 15.8 min); **(B)** GHK (96.3% pure, tr = 3.4 min); **(C)** F_4D-GHK (Pep2) (99.6% pure, tr = 14.3 min); and **(D)** F_4D-KHG (Pep3) (96.8% pure, tr = 14.2 min).

S2. Characterization of the assembled peptides

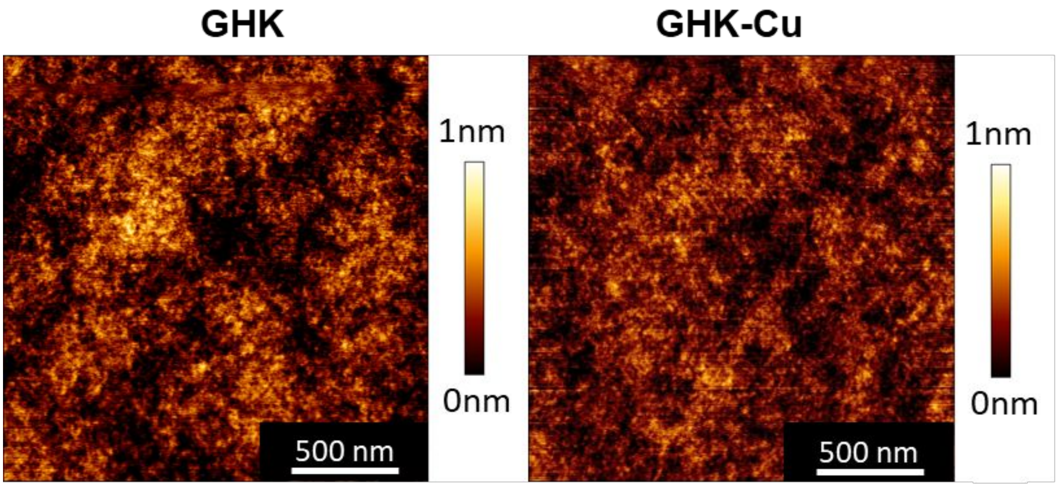

Figure S10. Representative AFM images of GHK and GHK-Cu revealing their inability to self-assemble, resulting in the absence of discernible nanostructures.

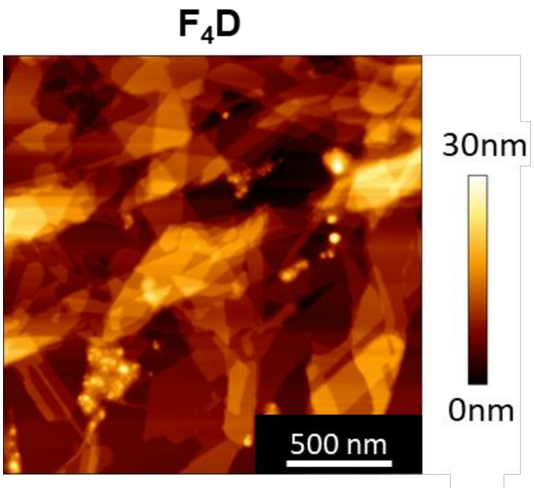

Figure S11. AFM images of the the nanostructures generated by F_4D in water.

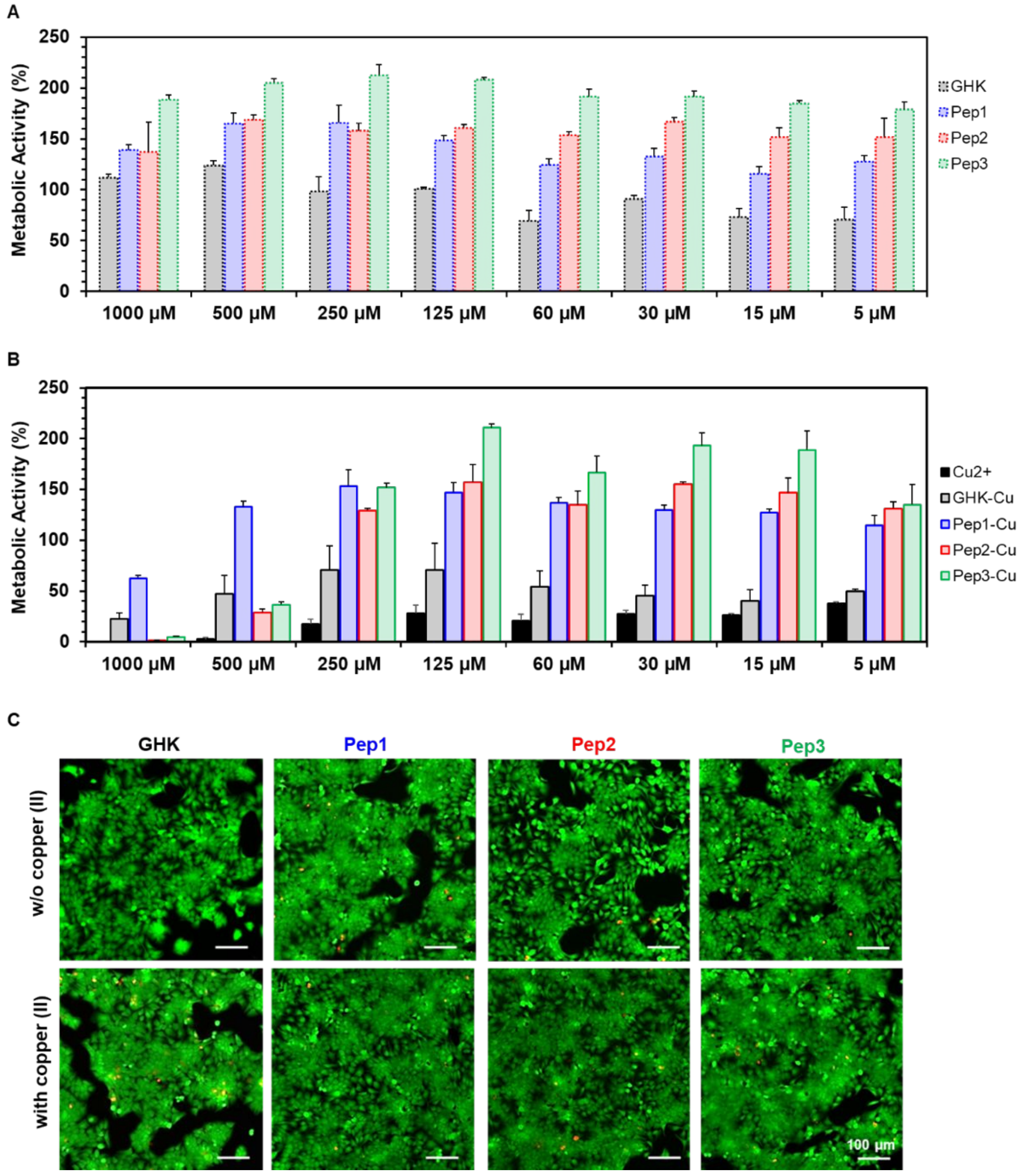

Figure S12. Assessment of the peptides cytotoxicity: HaCaT metabolic activity after 24h of incubation with: **(A)** only peptides at different concentrations and **(B)** peptides and Cu^{2+} at a 1:1 ratio. The presented values were normalized by the control experiment, i.e., HaCaT cells cultured in serum-free medium. **(C)** Live/Dead assay of HaCaT cells after 24h of exposure to 250µM of GHK and the different peptides, in the presence and absence of Cu^{2+}. Scale bar = 100µm.

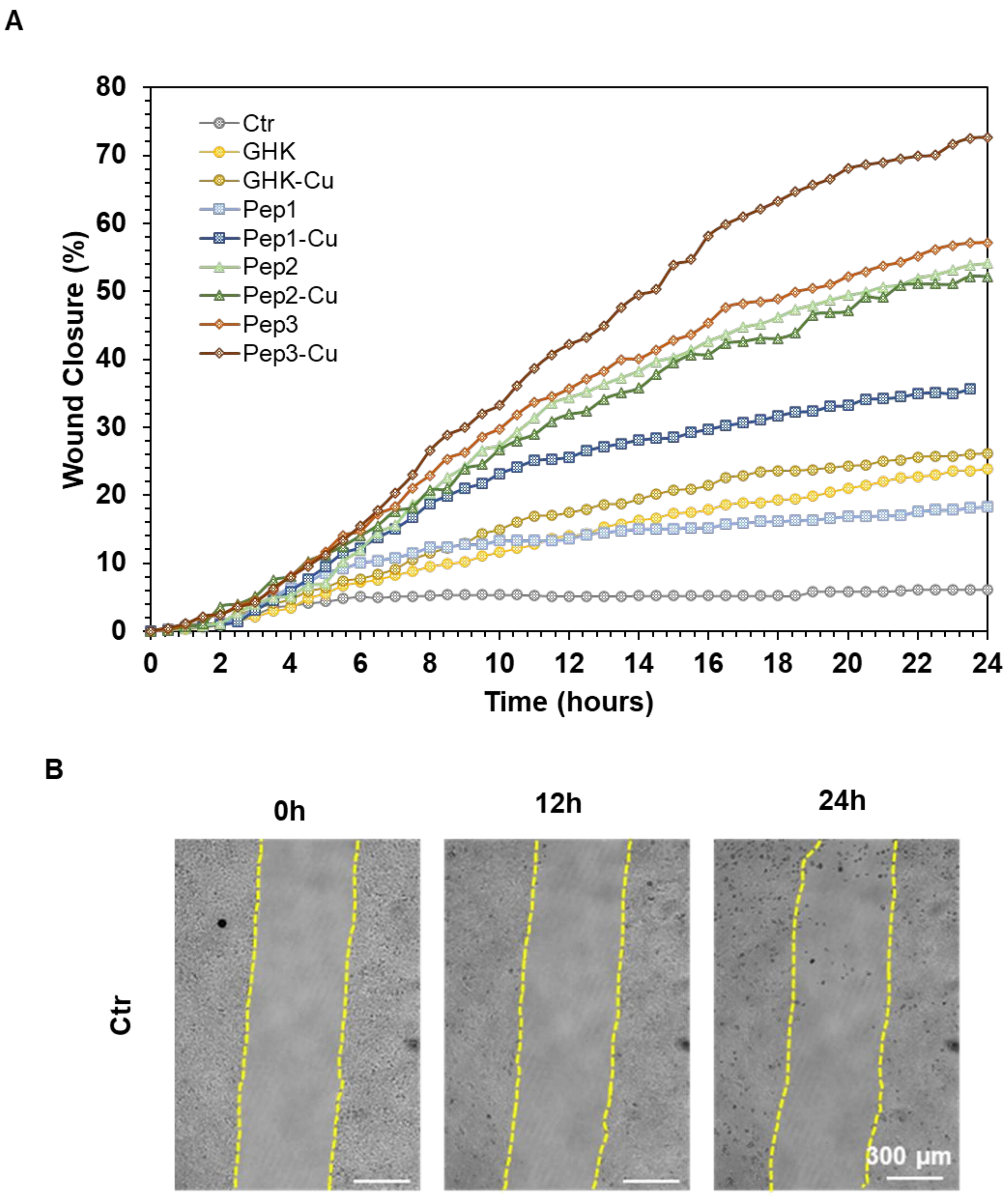

Figure S13. Scratch wound-healing migration assay: **(A)** Quantitative analysis of wound closure percentage by HaCaT cells exposed to 250µM of GHK, GHK-Cu, Cu-free supramolecular peptides (Pep1-Pep3) and Cu-complexed supramolecular peptides (Pep1-Cu, Pep2-Cu, Pep3-Cu). **(B)** Representative images of HaCaT cell migration in serum-free culture medium (control (ctr) experiment), scale bar = 300µm.

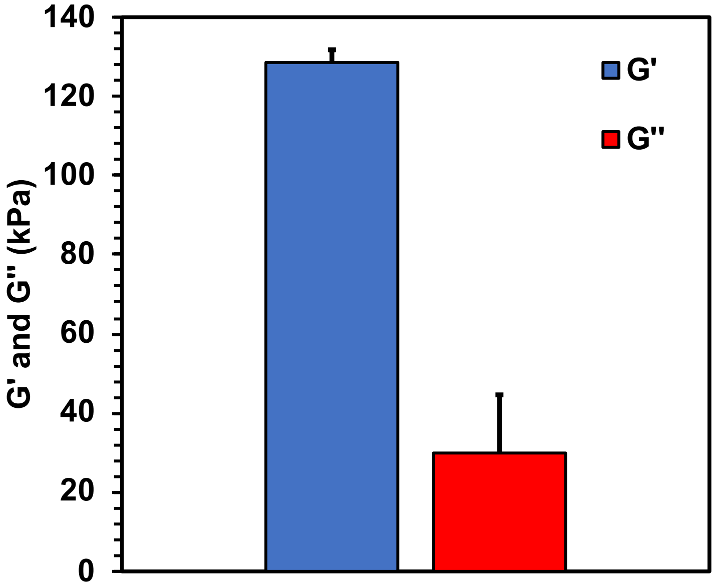

Figure S14. Elastic (G') and viscous (G'') modulus of the Pep3-Cu hydrogel determined by rheology.

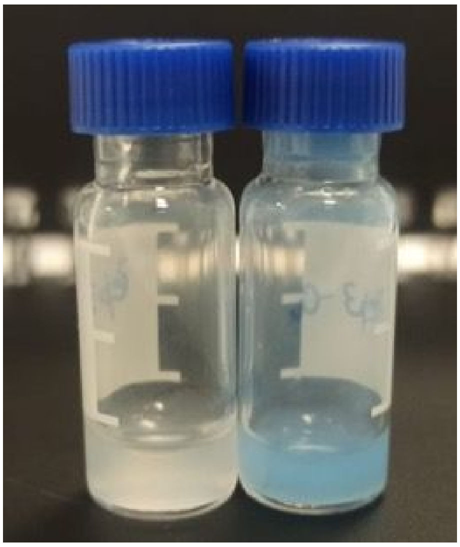

Figure S15. Photographs of the pre-gelation solutions of Pep3 (at a concentration of 30mM) in the absence (left) and presence (right) of $CuCl_2$. The blue color is indicative of the presence of Cu^{2+}.
